# Effects of Attitude, Motivation, and Eagerness for Physical Activity among Middle-Aged and Older Adults

**DOI:** 10.1155/2020/1014891

**Published:** 2020-08-27

**Authors:** Md Mizanur Rahman, Dongxiao Gu, Changyong Liang, Rao Muhammad Rashid, Monira Akter

**Affiliations:** ^1^School of Management, Hefei University of Technology, Hefei 230009, China; ^2^Key Laboratory of Process Optimization and Intelligent Decision-Making, Ministry of Education, Hefei 230009, China

## Abstract

**Background:**

Although physical activity (PA) is a noninvasive and cost-effective method of improving the quality of health, global statistics show that only a few middle-aged and older adults engage in the recommended PAs. This is due to a lack of motivation and companionship.

**Objective:**

This study analyses the attitudes and self-determined motivation of Chinese middle-aged and older adults for PAs and their eagerness to participate in PAs such as sports, exercise, and recreational and cultural activities (RCAs), from attitudinal, eagerness, and motivational objectives of PAs perspective.

**Methods:**

A cross-sectional study was carried out on 840 middle-aged (35–54 years) and older adults (55+ years). To determine their attitude, eagerness, and self-determined motivation for PA, we used attitudinal, Eagerness for Physical Activity Scale (EPAS), and Situational Motivational Scale (SIMS). The data were analyzed with SPSS 23.0.

**Results:**

The results show that 39.1% of the participants were not satisfied with PAs. Compared with females, males reported a less positive attitude towards PAs. Moreover, a positive attitude decreases with age. Participants' motivation and eagerness in activities such as RCAs, exercise, and sports are decreasing. Regarding self-determined motivation, there are gender differences in RCAs, but there is none for exercise and sports participation.

**Conclusion:**

The findings show the importance of RCAs and the support of family and friends enhancing the eagerness, attitude, and motivation to participate in PAs. Furthermore, the findings can help to create more effective PA programs for middle-aged and older adults. By engaging in RCAs, participants can reap the benefits of PAs. Participating in RCAs can lead to social equity in health.

## 1. Introduction

Globally, 13% of adults are obese, and 39% are overweight; these numbers are expected to increase in the coming years [1]. For instance, according to the World Health Organization (WHO), 36.2% of Chinese adults are overweight, and 5.9% are obese [2]. As they advance in age, adults face several health-related, debilitating diseases, such as diabetes, cardiovascular disease, and dementia. Despite the age-related changes, they need to maintain a good quality of health and life and improve their wellbeing. Recent studies show that when adults engage in PAs, it leads to health benefits, such as an increase in muscle power [3] and improvement in mental health, physical health, cognitive functions, and self-assurance. PAs decrease depression, anxiety, dementia, and coronary heart diseases [4]. According to Yu and Lin [5], “the specific type of PA (e.g., walking) itself may be a key in promoting PA for older adults and the general adult population” (p. 483). Others denote that PAs are connected with some sort of enjoyment [6], self-determined motivation [7, 8], and the fulfillment of basic psychological requirements. Several theories are used to guide, encourage, and enhance the participation of PAs and evaluate adults' active behaviors [9].

Several scholars have attempted to investigate the fundamental dynamics of the attitudes of participants of PAs [10, 11] and the benefits of PAs [3, 12, 13]. Only a few studies have examined the role of attitude and motivation in enhancing PAs participation. Attitude has been considered as an interrelated concept. In contrast, enjoyment [6] is regarded as an essential characteristic of self-determined motivation, which motivates people to engage in PAs [14]. Most studies used the self-determination theory (SDT) [7, 8] to examine people's approach to PAs by analyzing self-determined motivation [15]. SDT explains motivation and human behavior with a critical focus on the supply of a differentiated motivation approach.

Both controlled and autonomous motivations are distinguished by amotivation initiated and controlled by forces that are out of a person's internal control. Both motivations may be regulated to participate in individual activities, and they can also generate higher participation. Participants who are autonomously motivated show signs of satisfaction and developmental health [16]. The fundamental characteristic of SDT is the relationship between the amotivation and the joy of fulfillment [7]. Although many autonomously motivated participants do complete their essential requirements of PAs, there are a significant number of participants who want to support and improve their controlled motivation and minimize autonomous motivation [17, 18]. To increase PAs, we should decrease amotivation in the participants for them to focus on the improved level of satisfaction.

### 1.1. Attitudes towards PAs

Attitude can determine one's involvement in a specific behavior [11]. In PAs, positive disposition leads to a constructive attitude, while a negative disposition yields a destructive attitude. Studies about the attitude towards PAs [10, 19] have approached the idea from the positive perspective of enjoyment. This approach is vital in establishing optimistic involvements in PAs and hence promotes participation. In the current scenario, it will be significant to identify the relationship between attitude towards PAs and engagement in PAs.

### 1.2. Self-Determination and Attitudes towards PAs

According to the relational theories of human behavior development, participants' self-determination and positive attitude development depend on the PA's environments [20]. As middle-aged (35–54) and older adults (55+) carry traits of individualism, the organizing process should consider their aspiration and needs in the entire elements of PAs. Middle-aged and older adults' self-determined motivation and attitude towards PAs can be influenced by different variables, such as age, gender, family, and friends influence and attachment with cultural activities (dance, dance exercise, wushu (武术), tai chi chuan (太极拳), and qigong (气功)) [21–24]. In this perspective, attitude, motivation, and location of the activity play a significant role in gradually improving middle-age and older adults' level of participation in PAs.

### 1.3. Eagerness towards PAs

Eagerness is the way of recognizing behavior that influences participants to undertake a particular action that contrasts rationally or instrumentally driven practices. This idea is theoretically attached to live understandings [25] to represent the persons' situation and evaluation base when encountering new occurrences. Eagerness also indicates a regulatory tendency towards behaviors that evaluates personal importance or is in itself significant. Eagerness for PAs encourages participants' mental condition, enhances passion, and incredible longing or desire for PAs, which is good [25] for health. Hope is significant in understanding the participant's drive for learning and improvement [26]. Moreover, eagerness is associated with the encouragement of desirable behavior rather than the prevention of negative behavior [27]. In PAs, the concept of eagerness illustrates the motivation for a particular action, which is fulfilling and satisfying. Furthermore, the psychological qualities of eagerness towards PAs, which is hope and positive intention to maintain PAs in the future, possess significant potential in predicting sustainable involvement and participation in PAs. Researchers [25] show that eagerness for PAs has predictive validity above self-determined motivation.

As stated above, attitude, motivation, and eagerness for PAs are assumed to be relevant predictors of PAs. However, question such as “Do these factors motivate and facilitate middle-aged and older adults to engage in PAs [24]?” remains unanswered. Moreover, existing studies [24] lack the comprehensive theoretical representation of factors that could enhance participants' attitude, motivation, and their effects to increase their PA levels. Only a few studies identify the effects of attitude and motivation on PAs in adolescents. Therefore, it is vital to examine the effect of the support of family and friends on these middle-aged and older adults' attitudes, eagerness, and motivation for PAs. It is essential to consider how participants realize and adopt their family and friends' PA-related attitudes and behaviors in their values, understandings, and intentions to be physically active people. To bridge this research gap, we integrate PA constructs in different groups, such as sports, exercise, and RCAs with participants' enjoyment level of PAs, attitude, motivation, the support of family and friends, and eagerness for PAs. Hence, this study will examine the antecedents and the effects of attitude, motivation, support of family and friends, and eagerness of middle-aged and older adults for PAs.

By considering the points mentioned above, this study investigates the level of PAs in middle-aged and older adults in China. The objectives of this study are three-fold. The first is how the enjoyment of and assess to PAs affect the attitude of middle-aged and older adults towards PAs. The second is the participants' self-determined motivation and their eagerness to participate in different types of PAs, such as sports, exercise, and RCAs. The last objective is how participating in RCAs is correlated with sports and exercises, and how these three groups affect participants' motivation for PAs. Overall, this study also measures the relationship between the attitudes, self-determined motivation, and eagerness with actual involvement in PA.

## 2. Materials and Methods

### 2.1. Sample Selection and Data Collection

In the first part of the questionnaire, the participants were asked to state their gender, age, marital status, social environment motivator, and physical limitations. In the second part, we used the International Physical Activity Questionnaire (IPAQ) (In a typical week, how many hours do you spend participating in physical activity?) to identify participants' PAs levels. After that, we used the SIMS [28] and EPAS [25] questionnaires to determine participants' PA motivation and eagerness for PAs. Before the primary survey, we conducted a study based on a focus group of a few teachers and PhD students from the school of management who have specialized skills in survey design. Subsequently, based on the recommendations of the focus group, minor changes were made in the sequence and wording of some of the questions. Second, to get the feedback and confirm the content's validity with the participants' attitude, motivation, and eagerness for PAs, 40 valuable random sample reviews were analyzed. After calculating Cronbach's alpha value, we realized that the mean, standard deviation, and factor loading values were significantly high; hence, we did further investigation. After this analysis, we approved the final version of the questionnaire. The authors are fluent in English and Chinese. They translated the English version of the questionnaire to the local language (simplified Chinese) and translated it back to English to check the quality of the translation. This translation approach was adopted because of the recommendations of the translating committee [29]. After we made changes to some words, the questionnaire was in its final version. The final version of the questionnaires was sent to a different group of people via a web link and barcode. The questionnaires were administered in different places, such as playgrounds, parks, malls, and recreational centers, where middle-aged and old people engage in different kinds of PAs.

### 2.2. Participants

Self-administered questionnaires were used to collect the data from different big cities in China, such as Shanghai, Beijing, Hefei, Bozhou, and Guangzhou. The survey was conducted with the help of trained students who have experience in data collection. The main target population includes those who are close to parks, malls, playgrounds, and recreational centers. For wushu, tai chi chuan, and qigong, we collected the data from Huangshan International Wushu compaction and Bozhou International health Qigong Expo, 2019, and the 11^th^ Huatuo WuqinXi health and festival exchange completion. We targeted the middle-aged and older respondents; the justification for this choice is that these people prefer PAs. With the help of the researchers, the respondents were given a gift card (红包) after completing the survey. A total of 894 questionnaires were collected, and 54 of those were rejected based on principles and missing data. Hence, 840 questionnaires were used for the final analysis. The details of the demographics are shown in Table 1.

### 2.3. Measures

A structured questionnaire was designed, and we used Likert-type response formats. For attitude, motivation, and eagerness, we used a seven-point response that ranges from “strongly disagree” (1) to “strongly agree” (7). For Self-Determination Motivation Index (SDMI), we used a −18 to +18 range scale. For the total number of PAs, we used a six-point range; for the support of family and friends, we used a five-point response ranging from “strongly disagree” (1) to “strongly agree” (5). To keep a degree of rationality, we took the constructs from existing studies, especially the items for attitude and motivation dimensions were adopted from Guay et al. [28]. The elements for the participants' eagerness were adopted from Säfvenbom et al. [25]. The construct items of the total amount of PAs and the support of family and friends were adopted from Rahman et al. [24].

#### 2.3.1. Total Amount of PAs

Participants gave information about their weekly PA levels. PA levels were evaluated using the IPAQ [30], which is used to determine the weight of the PA and judgmental instrument that accounts for reliability and validity. To evaluate the total weekly level of PAs of the participants, we asked “In a usual week, in total, how many hours do you spend to participate in physical activity: 1-2, 3-4, 5-6, 7-8, 9-10, or 11 hours in each week?” [24] (p.3). The reactions were categorized into six different segments as 1, 2,…, 6 (see Table 2).

#### 2.3.2. Self-Determined Motivation

We used the Situational Motivation Scale (SIMS) [28], which contains 16 questions to assess the participants' self-determined motivation for PAs. Previous studies on PAs used SIMS as a reliable and suitable tool [25, 28, 31], which uses four subdimensions to assess and measure why participants engage in PAs. The subdimensions are (1) intrinsic motivation (I feel good when I engage in this activity), (2) identified regulation (I believe that this activity is good for me), (3) extrinsic motivation (this is something that I have to do), and (4) amotivation (I do this activity; however, I am not sure if it is worth it).

#### 2.3.3. Self-Determination Motivation Index (SDMI)

To determine the participants' PA position on the self-determination range, we used an SDMI, which was built from four subscales of SIMS. The SDMI illustrated the potency of the participants' self-determination, and it is expressed as follows:

(1) $$\begin{array}{c} \text{SDMI}=2\times \text{IM}+\text{IR}-\text{ER}-2\times \text{AM}. \end{array}$$

This SDMI uses simple weighting, and it ranges from −18 to +18, where a higher score indicates stronger self-determination.

#### 2.3.4. Support of Family and Friends for PAs

The support of family and friends for PAs was measured by five questions, for example, “How often do your family members or your friends inspire you to do PA, such as exercise and sports or participate in RCAs?” and “How often do you and your family members go out for PAs during holidays?” These were used to evaluate the perceived verbal and behavioral support of family and friends of middle-aged and older adults for engaging in exercise, sports, and RCAs in daily life and weekends.

#### 2.3.5. Involvement in PAs Contexts

Middle-age and older adults' PA levels were calculated for three groups of PAs. Group 1 (exercise) consists of structured and unstructured body movements, such as walking, jogging, cycling, running, weight lifting, and water aerobics. Group 2 (sports) includes participating in PAs taking place casually or in an organized manner, such as badminton, basketball, tennis, racquetball, table-tennis, soccer, and golf, to maintain and develop physical capabilities. Group 3 (RCAs) includes activities such as dance, dance exercise, wushu (武术), thaichi (太极), tai chi chuan (太极拳), and qigong (气功) [21, 22, 24].

#### 2.3.6. Eagerness for PAs (EPA)

EPAS [25], which is one-dimensional and containing nine questions, was used to assess the EPAs. EPAS items were used to evaluate four significant correlates: identity, emotional experience, cognitive evaluation, and behavior in participating in PAs, such as exercise, sports, and RCAs. The primary aim of using this scale is to estimate the affective and cognitive aspects of the participant's desire to be physically active, enjoy the activity, and self identity by engaging in a particular PA. We analyzed the behavioral issues, such as the intentions and expectations of the participant in keeping up with PAs (I enjoy keeping fit or being physically active).

### 2.4. Data Analysis

To check and measure the levels of PAs and the motivation to engage in them, we analyzed the data with SPSS 23.0. Statistical analysis was utilized to find out the preferred type of PA and determine what highly motivates middle-aged and older people to engage in PAs. To compute the participants' PAs levels, we conducted F-test and one-way ANOVA to assess the PAs groups (sports, exercise, and RCAs). The significance level for alpha was *α* ≤ 0.05. We also performed a post hoc Bonferroni test on a series of individual tests that compare the mean of each group to the mean of every other group [32]. We used the adopted editions of SIMS and EPAS to evaluate the participants' motivation and eagerness, respectively. We used multivariate regression analysis to predict motivation (SDMI) for PAs. The different types of PAs (sports, exercise, and RCAs) are the independent variables, and the SDMI scores are the dependent variables. This analysis enabled us to search for the outcome of a particular type of PA on the motivational scales. The results are considered significant when *p* ≤ 0.05.

## 3. Results

### 3.1. Common Method Bias

According to Mackenzie and Podsakoff [33], when data are gathered from only one source at the same time, the issue of common method bias may impact the validity of the study. To check the common method bias, we performed Harman's single-factor test. The outcome shows a value of 31.8%, which is below the cutoff rate. Thus, the outcome confirms that there is no severe concern about common method bias. We then checked the reliability of all the variables; the results were satisfactory.

### 3.2. Attitudes towards PAs

First, we used IPAQ to test the participants' PAs levels over the last seven days. The correlation assessments show that the IPAQ's short edition has a Cronbach's alpha (*α* = 0.83). We calculated the alpha (*α*) values for sports (*α* = 0.82), exercise (*α* = 0.84), and RCAs (*α* = 0.87) (Table 2). This research found that there is diversity in PA levels between sports, exercise, and RCAs. The PAs of the participants included sports (*n* = 132), exercise (*n* = 273), and RCAs (*n* = 435). The descriptive statistics of PAs involvement show that the participants perform vigorous PAs (*M* = 3.53) and moderate PAs (*M* = 3.84) every week.

Middle-aged and older adult's satisfaction in engaging in PA results is shown in Table 1. From left, Table 1 shows the average score of the respondents (5.60) (column 1). The results show that 11.1% of participants do not like PA (column 2), and 27% of the participants reported that it is challenging for them to engage in PAs (column 3). The results also show that 39.1% of the participants are not fully satisfied (column 2 plus column 3) with PAs; on the other hand, 61.9% of the middle-aged and older adults are satisfied with PAs (column 4).

Compared with older adults (*M* = 5.4), the middle-aged (*M* = 5.7) indicated a higher enjoyment level (*t* = 3.6; *p* < 0.001) and frequently engaged in PAs, with a chi-square *χ*^2^ = 44.7; *p* < 0.001.

The descriptive statistics show that the middle-aged and older female adult participants (*M* = 5.7) have the highest scores in the level of enjoyment (*t* = 5.6; *p* < 0.001). On the other hand, the male participants (*M* = 5.2) gave positive responses about PAs, with a chi-square *χ*^2^ = 114.2; *p* < 0.001. In addition, participants who engage in RCAs reported the highest score (*M* = 5.9) in PAs for enjoyment (*t* = 3.5; *p* < 0.001) as compared to participants who engage in exercise (*M* = 5.2), and sports (*M* = 4.8). One-way ANOVA shows the enjoyment level in PAs (*F* = 47.7; *p* < 0.001) for middle-aged and older adults. Post hoc Bonferroni test showed that participants that engage in RCAs outside the home have a higher score of enjoyment (*t* = 3.9, *p* < 0.001) in PA (*M* = 5.9) than those that participate in organized movement activities, exercise (*M* = 5.1) and sports (*M* = 4.8), as well as the lowest levels of enjoyment (*t* = 3.1, *p* < 0.001).

### 3.3. Self-Determined Motivation in PAs

The self-determined motivations for sports, exercise, and RCAs are *M* = 6.6, *M* = 7.0, and *M* = 7.6, respectively. A regression analysis (including variance) for eagerness and self-determined motivation for outdoor PAs as functions of age and gender was conducted. The descriptive statistics show gender differences for weekly PA levels and the subdimensions of the SDI.

The statistics also show strong relationships among RCAs, participants' involvement, support of family and friends, eagerness, the total amount of PAs, and motivation for involvement in different types of movement activities. The SDMI in PAs and all subdimensions of SDMI in PA are shown in Table 3.

Table 4 shows the multivariate regression analysis. The upper part shows the overall model explained by 40.6% of the total variance in SDI (*F* = 81.16; *p* < 0.001). According to this table, there are gender differences, but there is no significant difference in middle-aged and older adults. This table also shows that the model took into account the gender, age, the total amount of PAs, support of family and friends, and eagerness for movement activities. Participation in sports and exercise has no significant relationship between middle-aged and older adult's SDI in PAs, but participation in RCAs has significant results (*StB* = 0.114; *p* < 0.007).

The distinct subanalysis for genders showed an interaction outcome among participation in RCAs and gender's self-determined motivation to participate in PAs. The outcomes are displayed in the lower half of Table 4. The RCAs have a significant effect on male and female middle-aged and older adults' self-determined motivation for PAs; *StB* = 0.047; *p* < 0.031 and *StB* = 0.082; *p* < 0.025 for males and females, respectively. Furthermore, the descriptive analysis showed no significant differences in males (*M* = 7.1) and females (*M* = 7.2) and SDI scores between middle-aged and older adults (see Table 1). Moreover, the group that engages in sports has a lower SDI score (*M* = 2.5) than the score associated with exercise (*M* = 6.1). However, middle-aged and older adults who engage in RCAs as their major form of PA had significant scores on their SDI score (*M* = 9.5). For RCAs participants, there were differences in the average participation values for males (*M* = 5.6) and females (*M* = 7.6) with higher enjoyment level (*t* = 3.6; *p* < 0.009).

## 4. Discussion and Implications

Our results validate the existing body of literature [34–36]. The primary aim of this study is to assess the attitudes of middle-aged and older adults towards different groups of PAs. The effect of attitudes varies according to the type of activity, age, and physical condition. According to our analysis, there is a positive relationship between the participant's intentions and attitude towards PAs. Furthermore, the results show that attitude towards PA has high scores, which indicate that Chinese middle-aged and older adults have positive attitudes towards PAs. In particular, compared with males, middle-aged and older female adult participants have a more encouraging attitude towards PAs; this supports existing studies [34, 35]. Moreover, this study shows that middle-aged and older adults who do not participate in or enjoy movement activities or split their self-interest into competitive movement activities may take part in PAs. Still, in the long run, it can affect their PA involvement. Hence, they can develop a negative attitude towards PAs and eventually get demotivated to participate in PAs.

Individuals whose primary activities are RCAs have the highest proportion of PAs (Table 1). It emphasizes that more middle-aged and older adults participate in RCAs rather than in sports (51.8% vs 15.7%) due to a lack of enjoyment, health condition, and limited interest. Engaging in RCAs is highly correlated with motives related to social engagement and satisfaction. Moreover, middle-aged and older adults' attitudes and eagerness are critical in motivating them to engage in RCAs, but it is not that essential in exercise and even less in sports. Although PAs are fun and enjoyable, they are not without challenges. People willingly engage in activities with playfulness and thereby need little or no extrinsic motivations to do so. In particular, the activities involve enjoyment, which builds a sense of eagerness and creates an opportunity for social relations (RCAs and sports). The results of this study indicate that the participants might be eager to engage in PAs but do not yet get involved in competitive sports or exercise, due to some personal reasons like basic psychological needs. However, participants that engage in RCAs show more eagerness, and this increases a positive attitude towards participating in PAs to maintain better health.

The analysis of this study also validates earlier studies about the relationship between SDT and PA [21]. According to the SDT, the results of this study indicate that when participants are supported to feel autonomous, they are more likely to be intrinsically motivated. Supporting surroundings not only promotes their autonomous motivation but also positively increases their beliefs (attitudes) towards that behavior. There is no significant difference in the self-determined motivation index for PAs between male and female participants, but in the group activities, it shows great differences. Male participants' interest in sports or exercise and female participant's interest in RCAs reported considerably better scores on self-determined PA.

Furthermore, these findings highlight the significance of motivation, eagerness, and attitude towards RCAs in maintaining better health. Moreover, female participants show high levels of interest in PAs with RCAs but a lower level in sports, which supports earlier studies [37]. Moreover, this study shows that Chinese female participation in RCAs is a key predictor for middle-aged and older adults' participation in PAs. Finally, the results of the relationship between eagerness and amotivation show a negative association towards PAs, which is contrary to earlier studies [25]. This indicates that it is affected by demands, recommendations, and positive experiences. Therefore, participating in RCAs could be one of the critical predictors for middle-aged and older adults.

This study also investigates the relationship between the support of family and friends and participating in PAs. We found that the support of family and friends is associated with PAs; where friends' engagements in PAs influence them significantly because of a better source of gatherings and company [38]. As they advance in age, older adults' dependency increases. Hence, compared to friends, family members were a better source of social control (reducing risky health behaviors), as well as being instrumental and providing emotional support. We found that participants who regularly participate in PAs with friends or family members have more opportunities to achieve a high level of physical activeness.

### 4.1. Implications for Scholars

Based on the above discussion, this study provides some theoretical enlightenment. The results of this study confirm the importance of PAs in middle-aged and older adults by integrating people's motivation in the model to measure their intentions and actual attitude towards PAs. Moreover, this study also highlights why it is necessary to consider the effect of attitude and eagerness on the different dimensions of motivation when investigating people's motivation in the model to measure their intentions and actual attitude towards PAs. Our analysis shows that motivation, attitude, and eagerness play a significant role in developing people's attitude towards PAs.

Second, from the perspective of the role of PAs values, the findings of this study show that sports and exercise intention values have a profound influence on the participants' PA motivational intentions than RCAs values. These outcomes are contrary to those seen in existing studies [31]. Moreover, this study shows that RCAs have a significant direct influence on participants' age and gender.

Third, it is enjoyable to participate in RCAs, and this has a significant effect on PA's motivational intentions in middle-aged and older Chinese adults. A possible explanation for this could be that when participants find RCAs to be easy, they may develop a positive attitude for the effectiveness of the PA. We recommend that scholars apply these constructs in their research to get more awareness from their target audiences and add new facts to the PA styles.

Finally, RCAs are a new phenomenon in China, and they are studied along with demographic aspects such as gender, region (for example, urban or rural), and religion, which may affect its adoption. Researchers can develop mobile applications by using artificial intelligence digital tools (like a watch) to guide or monitor how people should engage in PAs to maintain better health in their everyday life. With these applications or tools, participants can ask any questions in their national language about a method without the difficulty of engaging in the activity.

### 4.2. Implications for Managers

This study also has several implications for project managers. First, project managers are strongly encouraged to improve their RCAs techniques [39] because this aspect is essential for participants who use RCA's platforms in their PAs. The participants are getting used to executing decisions on an “anywhere-anytime” basis, that is, whenever they get the time, they engage in PAs. For example, wushu and dance exercises have included structural assurances in their PA policy that has made PAs successful.

Second, the findings of this study offer handy information for PAs' decision-makers in China since the study provides full information on how to use different ways of managing and developing PA strategies. For example, this study confirms that RCAs have a significant influence on middle-aged and older adult's attitudes, eagerness, and motivations for PAs. The results show that participants of RCAs may be more likely to engage in PAs just for fun and enjoyment. In contrast, active participants may have a positive attitude and eagerness, which motivate them to use RCAs to enhance their psychophysical productivity. Thus, trainers or decision-makers should ensure quality and a diversified range of activities and services as well as other activities related to effective PA values. More precisely, given utilitarian PAs, project managers or decision-makers should offer multipurpose services and free training systems for participants. Moreover, due to cultural beliefs, it is easier for the Chinese to go outside and engage in PAs. RCAs, such as dance, wushu, and thaichi, provide a platform where Chinese can easily and conveniently engage in long-lasting PAs with all their norms and cultural values at any place of their choice.

Finally, it is vital for PA managers or decision-makers to adopt one of the most attention-grasping trends of PA, which is RCAs for Chinese. Chinese must understand the need to integrate people's PA systems before it is too late. It could assist people in developing optimistic views relating to PA values. This trend can reshape the PA sector.

## 5. Conclusions and Future Directions

The purpose of this study was to determine the attitude, motivation, and eagerness for different types of PA and how these groups of activities affect participants' motivation for PAs. This study confirms that gender differences play a vital role in shaping middle-aged and older adults' attitudes towards PA. The tendency of middle-aged and older adults' attitudes and eagerness for PAs declines with age. Participants' engagement in competitive, enjoyable PAs, such as RCAs, and motivation for PAs are the primary benefits of PAs. In RCA's participation, there are gender differences, where female participants significantly benefit. On the other hand, in sports, female participants' PAs show an opposite result. This study emphasizes the importance of middle-aged and older adults' engagement in PAs and seems to favor those participants' who are already engaged in cultural and aerobic activities, such as RCAs. Participants that engage in RCAs believe that these activities could be one of the sources of healthy behavior. Moreover, attitude and eagerness for PAs can be developed through the influence of family and friends. In addition, these will gradually improve middle-aged and older adults' attitudes, motivation, and eagerness; this might increase PA levels soon.

First, future studies can use cross-cultural data. Second, we only considered middle-aged and older adults; future studies can focus on young people. Finally, we used SPSS to analyze the data; future studies can use AMOS. Future research can study events, such as sports, exercise, and RCAs, in terms of thoughts as mastery, competence, and ability to participate and analyze how they are influenced and predicted by diverse motives and their role in improving health.

## Figures and Tables

**Table 1:** Demographics with immediate response to like/dislike of PA factor and participants reflection.

	Response rate	I do not like PA	I like PA, but it provided some difficulty	I like PA, and this should remain in the long run
	Mean (SD)		Percentage (participants)	
Total number (*N* = 840/894)	5.6 (1.4)	11.1 (93)	27.0 (227)	61.9 (520)
Males (*N* = 327/347) (38.9%)	5.2 (1.2)	11.9 (39)	34.2 (112)	53.9 (176)
Females (*N* = 513/547) (61.1%)	5.7 (1.4)	9.0 (46)	21.0 (108)	70.0 (359)
Age				
Middle age (35–54) (273/295) (32.7%)	5.7 (1.3)	8.8 (24)	34.1 (93)	57.1 (156)
Older adults 55+ (567/599) (67.3%)	5.4 (1.4)	12.0 (68)	21.9 (124)	66.1 (375)
Sports (132/140) (15.7%)	4.8 (1.2)	9.1 (12)	43.2 (57)	47.7 (63)
Exercise (273/291) (32.5%)	5.1 (1.3)	9.9 (27)	29.7 (81)	60.4 (165)
RCAs (435/463) (51.8%)	5.9 (1.4)	4.9 (21)	21.4 (93)	73.8 (321)

RCAs = recreational and cultural activities.

**Table 2:** Physical activity per week and other variables alpha values.

Constructs	Items	Hours/week	Sports (*n* = 132)	Exercises (*n* = 273)	RCAs (*n* = 435)	Cronbach's alpha (*α*)
(1) Physical activity per week	7	1–2	30	50	81	0.83
		3–4	61	95	123	
		5–6	23	75	135	
		7–8	12	39	66	
		9–10	4	12	23	
		≥11	2	2	7	
		*α*	0.82	0.84	0.87	
(2) Attitude towards PA	3					0.79
(3) Intrinsic motivation	4					0.87
(4) Identified regulation	4					0.93
(5) Extrinsic motivation	4					0.81
(6) Amotivation	4					0.82
(7) Family and friends support	5					0.78
(8) Eagerness of PA	9					0.95

**Table 3:** Mean and Standard Deviation of key study variables.

	Males	Females	Sports	Exercise	Cultural activities	Scale
Total number (*N* = 840)	327 (38.9)	513 (61.1)	132 (15.7)	273 (32.5)	435 (51.8)	
Males, % (*N* = 327)			102 (77.3)	183 (67.1)	42 (23.4)	
Females, % (*N* = 513)			30 (22.7)	90 (32.9)	393 (76.6)	
Middle age, % (35–54) 273 (32.5%)	115 (35.2)	158 (30.8)	81 (61.3)	87 (31.9)	105 (24.1)	
Older adults, % (55+) 567 (67.5%)	212 (64.8)	355 (69.2)	51 (38.6)	186 (68.1)	330 (75.8)	
Family and friends support, % (651; 77.5%)	253 (38.7)	399 (61.3)	99 (15.2)	210 (32.2)	342 (52.5)	
Friends and family support	3.5 (1.4)	3.8 (1.8)^*∗*^	3.4 (1.1)	3.8 (1.0)	4.0 (0.9)	1–5
Eagerness	4.8 (1.0)	5.2 (1.0)^*∗∗∗*^	3.2 (0.9)a	4.8 (0.8)a	5.7 (0.9)a	1–7
Intrinsic motivation (IM)	5.2 (1.0)	5.8 (0.9)^*∗∗*^	4.4 (1.0)a	5.2 (1.1)a	6.0 (0.9)a	1–7
Identified regulation-IR	5.1 (1.0)	5.7 (1.1)	4.5 (1.0)a	5.2 (1.1)a	5.9 (0.9)a	1–7
Extrinsic motivation (EM)	4.1 (1.2)	4.4 (1.1)^*∗*^	4.6 (1.0)b	4.5 (1.2)a	4.2 (1.2)ab	1–7
Amotivation (AM)	2.6 (0.8)	2.4 (0.9)	3.2 (0.9)a	2.5 (1.0)a	2.1 (0.9)a	1–7
SDMI in PA	7.1 (2.9)	7.2 (2.8)	2.5 (2.3)a	6.1 (2.9)a	9.5 (1.9)a	−18-18
Total amount of PA	3.4 (2.3)	3.8 (2.5)^*∗∗∗*^	2.2 (1.5)a	3.6 (1.8)a	4.5 (2.2)a	1–6

SDMI = 2 × IM + IR − ER − 2 × AM; Here, a, b, and ab = homogenous subsets indicate significant differences, using one-way ANOVA test, Bonferroni post hoc test (*p* ≤ 0.05). ^*∗*^*p* < 0.05; ^*∗∗*^*p* < 0.01; ^*∗∗∗*^*P* < 0.001.

**Table 4:** Multivariate regression analysis for predicting motivation (SDMI) in PA.

Coefficients *a*					
Model	Unstandardized coefficients		Standardized coefficients	*t*	Sig.
	B	SE	B		
Overall model: *R2* = 0.406; *F* = 81.16; *p* < 0.001					
(Constant)	0.249	0.524		0.475	0.635
Gender	0.321	0.164	0.054	1.957	0.051
Middle-aged and older adults	−0.554	0.165	−0.090	−3.350	0.001
Friends and family support	0.473	0.083	0.194	5.673	0.000
Total amount of physical activity	−0.230	0.064	−0.113	−3.581	0.000
Eagerness	1.300	0.074	0.540	17.589	0.000
Sports participants	0.096	0.240	0.012	0.400	0.689
Exercise participants	0.082	0.174	0.014	0.469	0.639
Recreation and cultural activity participants	0.185	0.068	0.114	2.727	0.007

Model male: *R2* = 0.485; *F* = 42.9; *p* < 0.001					
(Constant)	0.775	0.888		0.873	0.384
Middle-aged and older adults	−0.639	0.285	−0.092	−2.237	0.026
Friends and family support	0.645	0.149	0.263	4.319	0.000
Total amount of physical activity	−0.460	0.194	−0.142	−2.368	0.018
Eagerness	1.586	0.115	0.642	13.766	0.000
Sports participants	0.007	0.353	0.001	0.021	0.633
Exercise participants	−0.372	0.302	−0.058	−1.230	0.220
Recreation and cultural activity participants	0.190	0.087	0.047	1.036	0.031

Model female: *R2* = 0.346; *F* = 38.1; *p* < 0.001					
(Constant)	2.039	0.634		3.215	0.001
Middle-aged and older adults	−0.565	0.203	−0.100	−2.778	0.006
Friends and family support	0.486	0.114	0.201	4.255	0.000
Total amount of physical activity	−0.180	0.068	−0.107	−2.647	0.008
Eagerness	1.156	0.112	0.492	10.320	0.000
Sports participants	−0.436	0.329	−0.050	−1.323	0.186
Exercise participants	−0.074	0.224	−0.013	−0.332	0.740
Recreation and cultural activity participants	0.201	0.120	0.082	1.677	0.025

^a^Dependent variable: SDMI.

## Data Availability

Data will be available on request.
